# Stakeholder perspectives informing the design and development of a digital self-help intervention for healthy aging and mental well-being among older adults in urban India: a qualitative study

**DOI:** 10.1186/s12877-026-08107-0

**Published:** 2026-08-18

**Authors:** Indranil Saha, Devi Das, Susmita Dutta, Suchismita Hoda, Madhurima Khasnobis, Shraddha Sharma, Jeevan J., Treasa Lisbeth Sajan, Surajit Das, Neha Dahiya, Asim Saha, Lakshmanan Sethuraman, Arun Kandasamy, Neda Agahi, Nitya Jayaram-Lindström, Martin Kraepelien, Christopher Sundström, Amit Chakrabarti

**Affiliations:** 1 Department of Epidemiology, ICMR-National Institute for Research in Bacterial Infections, Indian Council of Medical Research, Kolkata, West Bengal India; 2 Department of Medical Research, Academy of Scientific and Innovative Research (AcSIR), Ghaziabad, Uttar Pradesh India; 3 Department of Mental Health, ICMR-Centre for Ageing and Mental Health, Indian Council of Medical Research, Kolkata, West Bengal India; 4 Department of Psychiatry, NIMHANS, Bengaluru, Karnataka India; 5 Division of Mental Health, ICMR- Headquarter, New Delhi, India; 6 Centre for Addiction Medicine, NIMHANS, Bengaluru, Karnataka India; 7 Department of Neurobiology, Care Sciences and Society, Karolinska Institutet, Stockholm, Sweden; 8 Department of Clinical Neuroscience, Centre for Psychiatry Research, Karolinska Institutet, Stockholm, Sweden

**Keywords:** Healthy Aging, Mental Health, Aged, Qualitative Research, Digital Health, Preventive Health Services, India

## Abstract

**Background:**

India’s older adult population is rapidly increasing, accompanied by a growing burden of common mental health problems such as depression, anxiety, sleep disturbances, and cognitive concerns. Promoting healthy aging and preventive mental healthcare has therefore become an important public health priority. Digital health interventions have emerged as promising approaches for improving access to preventive and supportive care among older adults. This study gathers perspectives from older adults, caregivers, and experts to develop content for a self-help digital intervention for older adults (≥ 60 years) in India, with a focus on healthy aging and the prevention of common mental health problems.

**Methods:**

A qualitative study, following the COREQ guideline, was conducted from January through September 2024 at two sites in India. Participants included community-dwelling older adults aged ≥ 60 years and their caregivers, as well as experts from diverse medical and allied health disciplines. Data were collected using FGDs, IDIs, KIIs, and free-listing. All interviews were audio-recorded, transcribed verbatim, and analyzed using thematic analysis. Transcripts were systematically coded, and codes were grouped into themes across predefined analytical domains.

**Results:**

Regular sleep routines, a balanced diet, and physical activity and exercise were considered beneficial for healthy aging. Furthermore, stress management, steering clear of negative thoughts, and practicing meditation were cited as essential for optimum mental health. Health management tips, exercise tutorials, dietary guidelines, mental healthcare advice, and sleep hygiene tips are expected in digital interventions.

**Conclusions:**

The findings suggest that digital interventions tailored to the sociocultural needs of older adults may play an important role in promoting healthy aging and supporting mental well-being in India. Stakeholder-informed digital modules addressing physical health, sleep, emotional well-being, social connectedness, and self-care practices may enhance engagement, accessibility, and preventive mental healthcare among older adults. Future research should evaluate the effectiveness, acceptability, and long-term sustainability of such interventions in larger and more diverse populations.

**Supplementary Information:**

The online version contains supplementary material available at 10.1186/s12877-026-08107-0.

## Background

The number of older adults has been increasing globally over the years [1]. According to the 2023 India Aging Report by the United Nations Population Fund (UNFPA), India’s elderly population is currently growing at a decadal rate of 41% [2]. By 2050, the elderly population is expected to constitute more than 20% of India’s total population, effectively doubling their current proportion [2]. In 2020, the World Health Organization (WHO) launched the Decade of Healthy Aging to address challenges associated with population aging. The United Nations Decade of Healthy Aging (2021–2030) recognized its significant impact beyond healthcare systems [3]. This initiative promotes practices that enhance autonomy, productivity, and overall quality of life for older individuals, including the mental, cognitive, and social dimensions of older adults’ well-being [4].

According to the WHO, healthy aging is “the process of developing and maintaining the functional ability that enables well-being in older age” [5]. Healthy aging includes preserving physical and cognitive functions, remaining socially active, and maintaining a positive mindset, incorporating overall well-being, mental health, and social involvement [6, 7]. As a result, older individuals will be able to enjoy longer, healthier lives with decreased morbidity and disability. Encouraging healthy aging at the population level is essential, as it can improve the quality of life for older adults, support their social engagement, and reduce the burden on healthcare systems.

A recent systematic review & meta-analysis revealed higher rates of sleep, anxiety, and substance use disorders among older adults in Asia. The findings highlight the significant impact of mental health disorders among older adults in lower middle-income countries (LMICs), underscoring the importance of strategic management [8]. It has also been noted that older adults in LMICs encounter considerable mental health issues because of limited access to conventional healthcare services [9]. The National Mental Health Survey of India indicated that older adults demonstrate a greater prevalence of depressive disorders and overall psychiatric conditions than younger adults do [10]. Prevalence of mental illness was found to be 43% among residents of old age homes [11]and another study among rural residents found it to be 33.9% [12]. Common mental disorders among this demographic include dementia and mood disorders, especially depression. Additionally, frequently observed conditions are anxiety disorders, substance abuse (both drugs and alcohol), delirium, and psychosis [13]. Studies emphasized the need for increased focus on detecting and preventing late-life psychiatric disorders [14].

The promotion of mental health and prevention of disorders in older adults emphasizes the importance of prioritizing healthy aging [15]. Recently, there has been a remarkable rise in digital health interventions that show promise in preventive care for older adults. In Western countries, digital healthcare has been found to promote healthy aging through improved access, monitoring, and self-care [16]. Forms of digital tools (e.g., internet-delivered with therapist support, app-based self-care) have also demonstrated the potential to manage mental health issues such as depression and anxiety in controlled clinical trials [17]. In India, during the COVID-19 pandemic, healthcare provided digitally served as a safer substitute for in-person therapy [18]. Digital interventions alleviate the shortage of qualified medical professionals, improve access, and reduce the cost of in-person care. They have also enhanced and expedited the delivery of care when face-to-face treatment is restricted [19]. With advancements in technology, digital health interventions empower older adults to actively manage their health, thereby facilitating early detection, prevention, and the delivery of personalized care as conventional medical practices transition to remote approaches [20–22].

The scope of this part of the Indo-Swedish collaborative research project was to explore stakeholder-identified needs and contextual factors related to healthy aging and mental well-being among older adults (≥ 60 years) in India, with the objective of informing the development of a digital self-help intervention. The study drew on perspectives from key stakeholders, including older adults, their caregivers, and relevant experts, to ensure that the intervention design reflected lived experiences, caregiving contexts, and professional insights. The study was guided by the following research questions: *(a) How do older adults*,* caregivers*,* and experts conceptualize healthy aging and common mental health disorders in the Indian context? (b) What barriers and facilitators to good mental health and healthy aging are perceived by key stakeholders? (c) What expectations*,* preferences*,* and requirements do stakeholders identify for a digital self-help intervention for older adults in India?* By systematically examining these perspectives, the study generated evidence to support the development of a contextually grounded and user-informed digital intervention for older adults in India.

## Methods

### Study design and settings

This study was guided by the World Health Organization’s Healthy Aging [23]. A qualitative descriptive approach was used to explore the perspectives of older adults, caregivers, and health-care experts regarding healthy aging, mental health needs, and expectations from digital self-help interventions in the Indian context [24]. Guided by an interpretivist orientation, the study aimed to understand participants’ lived experiences and sociocultural perspectives [25]. The stakeholder-centered co-design approach ensured active incorporation of stakeholder inputs to improve the relevance, acceptability, and usability of the proposed intervention [26].

This study was conducted from January to September 2024 at two sites in India. ICMR-Centre for Ageing & Mental Health (ICMR-CAM), Kolkata; National Institute of Mental Health and Neuro Sciences (NIMHANS), Bangaluru; & Department of Computer Science & Engineering, Calcutta University are research partners in India. The Department of Clinical Neuroscience, Centre for Psychiatric Research, Karolinska Institutet (KI), Stockholm and Stockholm Healthcare Service are research partners from Sweden. In Kolkata, beneficiaries (older adults aged ≥ 60 years and their caregivers) were selected from ward number 57 of Kolkata Municipal Corporation, whereas in Bangalore, they were selected from Karnataka Pensioners Association & Elderly Care centers. Both study sites were purposively selected based on prior community-based research conducted by these two institutes. The experts were selected from medical college hospitals. The reporting of this qualitative study followed the Consolidated Criteria for Reporting Qualitative Research (COREQ) guideline, and the completed checklist is provided in Supplementary Table 1 [27].

### Clinical trial number

CTRI/2022/10/046361.

### Study participants

Older adults aged ≥ 60 years and their caregivers were recruited as beneficiaries. Access to the target population was facilitated through prior community-based activities and collaborations established by the participating institutes within the study settings. Potential participants were approached during community visits and through local elderly associations and community networks. Participants were recruited from the community using a combination of criterion, purposive, and non-proportional quota sampling methods. Criterion sampling was used to select older adults who used smartphones in their daily lives and the caregivers who provided routine care and support to older adults. Purposive sampling was employed to include participants (both older adults and caregivers) who could provide rich, relevant, and diverse information regarding our research questions. Non-proportional quota sampling ensured representation across key strata, including age group (younger and older caregivers), gender (male and female older adults and caregivers), and socioeconomic status (upper-middle and lower-middle groups of older adults and caregivers).

Data were collected through focus group discussions (FGDs) and in-depth interviews (IDIs). At the Kolkata site, FGDs were conducted with 48 older adults across eight groups, with six participants in each group (four male-only and four female-only groups). In addition, two FGDs were conducted with 12 caregivers, comprising six participants per group (one male-only and one female-only group). Three caregivers (males) at the Kolkata site also participated in IDIs (Table 1). At the Bangalore site, FGDs were conducted with 48 older adults across eight groups, again with six participants per group (four male-only and four female-only groups). Two FGDs were conducted with 16 caregivers at this site, with eight participants in each group (one male-only and one female-only groups) (Table 1). FGDs were conducted separately for male and female participants to minimize the potential for power dynamics and social inhibitions that may arise in mixed-gender groups, thereby encouraging participants to share their perspectives freely. This approach also allowed the exploration of gender-specific viewpoints that might otherwise be underrepresented in mixed discussions. In total, the study included 127 beneficiaries, comprising older adults and their caregivers across both sites. All interviews were conducted in the local language to facilitate better understanding, promote open communication, and capture culturally relevant insights.

**Table 1:** Distribution of the different qualitative techniques employed at the two sites

Study participants	Study techniques (number)	Institute	Number
Beneficiaries (i.e., older adults aged 60 years or above, caregivers)	FGD (*n* = 20)	Kolkata	10
		Bangalore	10
	IDI (*n* = 3)	Kolkata	3
		Bangalore	0
Service providers/related stakeholders (i.e., geriatric specialists, psychiatrists, nursing personnel, psychologists, geriatric physical medicine specialists, and general physicians)	Free listing (*n* = 28)	Kolkata	13
		Bangalore	15
	KII (*n* = 22)	Kolkata	12
		Bangalore	10

Experts included key professionals involved in geriatric care and mental health services, namely geriatric medicine specialists, psychiatrists, psychologists, nursing professionals, physical medicine and rehabilitation specialists, neurologists, and general physicians. They were recruited from both study sites using purposive sampling to obtain multidisciplinary perspectives on healthy aging, mental health needs. Key informant interviews (KIIs) and free-listing were conducted with the experts in English. At the Kolkata site, 13 experts were approached to participate in KIIs, of whom 12 consented to participate (Table 1). At the Bangalore site, 15 experts were approached, and 10 participated in the KIIs. Experts who did not participate in the KIIs contributed through free-listing exercises, along with those who participated in the interviews (Table 1).

**Fig. 1:**
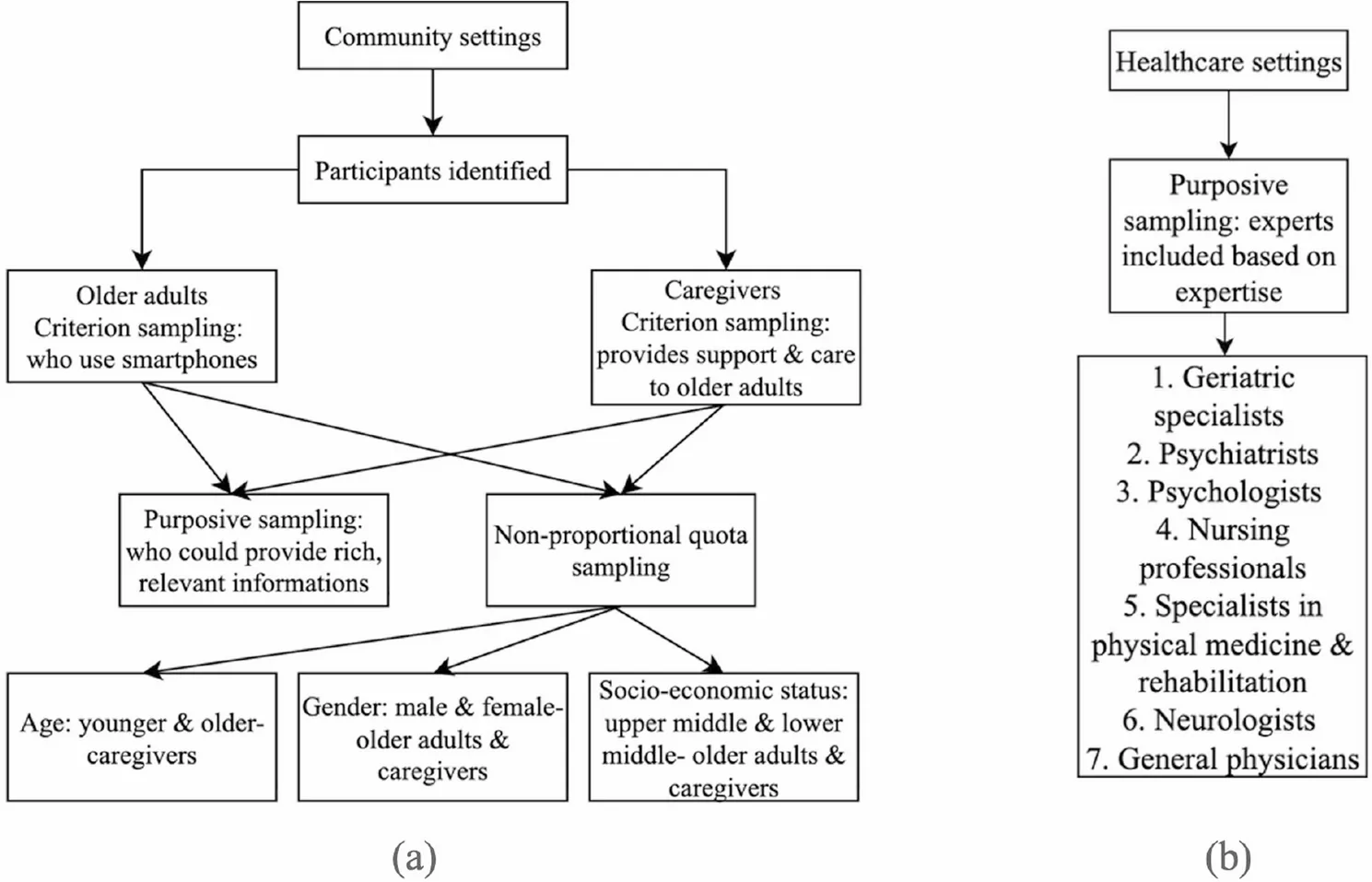
Flowchart illustrating the recruitment process of the (**a**) beneficiaries (older adults & their caregivers) from the community settings and (**b**) the experts from healthcare settings

The entire recruitment process of older adults, caregivers and the experts is illustrated in the flowchart (Fig. 1). Written informed consent was obtained from all participants prior to enrolment after they were informed about the objectives, purpose, and procedures of the study. Participation in the study was entirely voluntary, and participants were informed of their right to withdraw from the study at any stage without any consequences. Participants did not receive any monetary compensation for participation; however, refreshments were provided during the FGDs, IDIs and KIIs.

### Research team, interview guide, and data collection

The research team comprised employed project researchers (DD, SD, SH, MK, SS, JJ, TS) who conducted all FGDs, IDIs, and KIIs. All team members held postgraduate qualifications and formal training and prior experience in qualitative research methods, including conducting interview, facilitating group discussions and qualitative data analysis. All researchers were female except JJ and had prior experience in conducting FGDs, IDIs, and KIIs. Potential biases were mitigated through supervision by established researchers (IS, AC, AK, LS), who also have extensive experience and publication record in qualitative research, along with ongoing reflexivity to consciously identify and manage researchers’ preconceptions during data collection and analysis. The researchers shared the local language (Kolkata: *Bengali*; Bangalore: *Kannada*) and cultural background of the participants, enabling open communication during interviews and discussions. Although rapport was established during data collection, no prior personal relationship existed between the researchers and the participants before recruitment. Professional boundaries were maintained throughout the study to minimize potential researcher influence during interviews and discussions.

The FGD, IDI, and KII guides were developed specifically for this study, based on the available literature and evidence, to ensure objectivity and minimize personal assumptions affecting data collection and analysis (Supplementary Table 2) [28–34]. To ensure the validity of the content, the guides were reviewed by an external expert in qualitative research. Subsequently, the guides were pilot-tested under field conditions, and necessary modifications were made based on the pilot findings. The revised guides were then re-evaluated and formally validated by the same qualitative research expert prior to finalization. The semistructured guides included open-ended questions and flexible probing to explore participants’ perspectives in depth. An external expert in qualitative research also validated the free listing question.

FGDs were conducted with participants seated in a circle in a face-to-face format and facilitated by a trained moderator, supported by a note-taker, using a FGD guide. IDIs were conducted one-on-one with caregivers [35, 36]. KIIs with experts were also conducted individually using a KII guide. All FGDs, IDIs, and KIIs were audio-recorded with prior permission from the participants, and detailed field notes were taken during the interviews. In addition to verbal responses, researchers created sociograms to document group dynamics and interaction patterns, supporting accurate interpretation of the discussions. Attention was also given to areas of agreement, disagreement, and shared experiences alongside emotions that emerged during group interactions.

Each IDI and KII lasted approximately 40–50 min, while FGD lasted about 50–60 min. Interview transcripts were reviewed and discussed among the investigators on an ongoing basis to assess data saturation, and no follow-up interviews were conducted. Data saturation was considered achieved when successive FGDs, IDIs, and KIIs yielded no new codes, themes, or conceptual insights, as determined through the research team’s ongoing review of transcripts. In free-listing exercise, the experts were asked to list factors related to healthy aging in descending order of perceived importance [37].

### Trustworthiness of the study

Privacy and confidentiality were maintained during data collection, creating a comfortable environment that encouraged participants to express their views freely without hesitation. Researchers used empathetic listening, informal introductory conversations, reassurance of confidentiality, and flexible probing techniques to encourage participation and address communication difficulties or hesitations during interviews. Potential group discussion biases were minimized through skilled moderation, neutral probing, balanced group composition, and the inclusion of individual interviews to ensure diverse and independent responses. The collected data were debriefed to the participants for approval at the end of each FGD/IDI session. After the transcript, all the KIIs were sent to the concerned stakeholders for their concurrence and approval of their statements via email. The collected data were transcribed, translated, coded, and analyzed to maintain accuracy and consistency in translation. Additionally, the researchers reviewed the analyzed findings to detect any inconsistencies [38]. Methodological triangulation (FGDs, IDIs, KIIs, and free-listing) and data-source triangulation (older adults, caregivers, and experts) were employed to enhance the credibility and trustworthiness of the findings.

### Data analysis

Initially, the audio files were transcribed verbatim with the aid of field notes and then translated from the local language (Kolkata: *Bengali*; Bangalore: *Kannada*) into English for consistency (for FGDs and IDIs). All study data were managed and organized using Microsoft Excel (Microsoft Corporation, Redmond, WA, USA). Data analysis was conducted using six-phase thematic analysis framework proposed by Braun and Clarke [39], including familiarisation with the data, systematic coding, development and refinement of themes, defining and naming themes, and producing the final analytic narrative supported by representative quotations in line with the study objectives.

A hybrid thematic analysis approach, combining inductive and deductive methods, was employed. The inductive component allowed themes to emerge directly from participants’ narratives without imposing predetermined assumptions. This ensured that the analysis remained grounded in the real-world experiences, perceptions, and needs of older adults and caregivers, which was central to the study’s objective of understanding mental health needs and contextual factors influencing healthy aging. The deductive component enabled the organization and interpretation of these emergent themes within predefined domains aligned with the study objectives, namely healthy aging, mental health, and digital intervention. This approach allowed the study to capture unanticipated insights while maintaining analytic coherence and alignment with the intended application of the findings in digital intervention design.

The cognitive map was created with the help of codes, categories, and themes for each domain [40]. Data coding and thematic analysis were conducted independently by two researchers from each site, with regular team discussions to refine codes, resolve discrepancies, and finalize themes. The data generated from free listing underwent thorough cleaning procedures involving multiple iterations. Initially, one researcher grouped grammatical forms of identical words. The synonyms were subsequently consolidated in a second round, followed by grouping words representing analogous concepts in a third round. The salience of each item in every list provided by the participants was calculated. Thereafter, the free list salience of an item (Smith’s S) was computed by summing the salience of each word [37]. The data from the free list were used to construct a network analysis diagram to illustrate the co-occurrence of key factors contributing to healthy aging.

## Results

In this study, the ages of the older adults and caregivers ranged from 60 to 78 years and 28–59 years, respectively. At the Kolkata site, the mean age of the older adults was 65.63 ± 5.06 years, while that of the caregivers was 47.64 ± 8.51 years. The distribution of participants by sex was almost equal (51.2% male and 48.8% female). The participants in the FGDs and IDIs included individuals with various educational levels, ranging from graduate to postgraduate levels. It comprises people from all spheres of life, including homemakers, retired individuals, service and business personnels and self-employed individuals. The participants were mainly of upper-middle or lower-middle socioeconomic status.

### Perspective of the beneficiaries

#### Perception of healthy aging

All the older adults viewed healthy aging to be a holistic construct related to both physical and mental well-being, and particularly the absence of significant illnesses. Four key sub-themes emerged:

##### Physical health and functional independence

Most participants emphasized the need for maintaining a regular sleep routine, adhering to a balanced diet, participating in physical activity and exercise such as walking, yoga, light exercises and the ability to perform daily activities.

##### Mental and emotional well-being

A few participants described mental well-being as freedom from stress, avoidance of negative thoughts, being emotionally well balanced. Practicing meditation, prayer were perceived as vital for mental health.

##### Social connectedness and family support

**Fig. 2:**
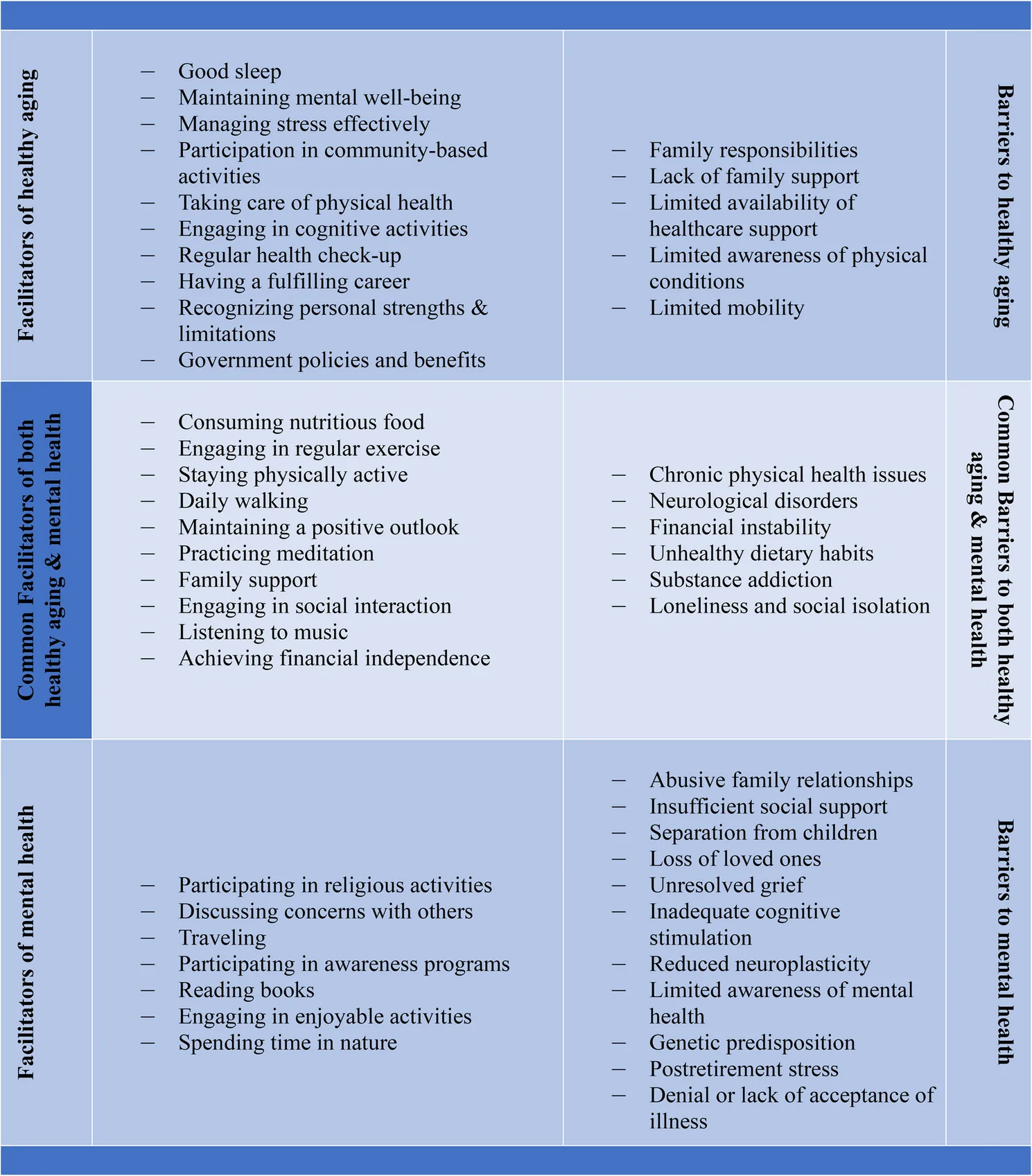
Facilitators and barriers to healthy aging and mental health among older adults as perceived and opined by beneficiaries, caregivers, and stakeholders

Most participants highlighted robust family support, positive intergenerational relationships, active community involvement, social connections as fundamental elements for emotional security and a sense of belonging in later life (Fig. 2).

##### Financial security and autonomy

Financial stability, including pensions or family financial support, was considered important for accessing healthcare, maintaining independence during aging.

*“To me*,* healthy aging means taking care of both my body and mind so I can avoid serious illnesses. With proper self-care*,* age is merely a number.”* Opined a 62-year-old female.

#### Practices adopted to promote healthy aging

Participants described various practices to maintain health, promoting healthy aging, which were categorized into the following sub-themes:

##### Lifestyle and health promoting behaviours

When asked about the practices adopted to promote healthy aging, most common practices found were engaging in physical activities such as walking, meditation, avoiding unhealthy foods.

##### Role of caregivers in health maintenance

Caregivers reported encouraging healthy behaviours, facilitating medical consultations, monitoring medication adherence, and supporting participation in social and religious activities.

#### Barriers to healthy aging

Multiple interconnected barriers to healthy aging came out from the FGDs and IDIs, forming several sub-themes:

##### Physical and health-related constraints

Majority of the participants reported health related conditions such as diabetes, cardiovascular disease, neurological issues, trembling hands and feet, body pain induced limited mobility hindered physical activity and thus affect the healthy aging process.

##### Psychological and emotional challenges

Majority of the older adults reported loneliness, anxiety, low mood, forgetfulness, fear of dependency, and sleep disturbances, often intensified declination of the physical health.

##### Social isolation

Older females experience higher levels of social isolation and spend considerable time alone, which significantly impacted their mental health (Fig. 2). Men reported emotional distress linked to reduced authority, financial independence, separation from children and perceived loss of respect within the family.

##### Environmental and infrastructural barriers

Caregivers highlighted unsafe and congested roads, lack of pedestrian pathways, and poor neighborhood infrastructure, which restricted outdoor mobility and discouraged physical activity.

##### Financial constraints

Some participants reported limited income post-retirement and rising healthcare costs, along with restricted access to nutritious food, medical care, and recreational activities, all of which negatively affected their overall well-being.

*“I have osteoarthritis*,* so walking and climbing stairs is hard now. I also feel tired in the mornings*,* making it tough to go for walks. Being retired means that my income is limited*,* so I cannot always follow all the dietary rules. My heart problem makes things even harder. Even with these challenges*,* I’m doing my best to adjust and keep going. Family responsibilities also make it difficult to focus on my health*,* but I’m trying.”* Reported by a 60-year-old male.

#### Changing role of older adults in the family

Shifts in family roles emerged as one of the major themes, with the following sub-themes:

##### Declining decision-making responsibilities

Majority of the male participants reported a perceived loss of authority and involvement in household decisions, which negatively affected their self-esteem.

##### Role transitions following widowhood or living separately

Widowed females or those living apart from children often assumed multiple roles, managing both household and external responsibilities, sometimes leading to physical and emotional strain.

*“I feel like my importance in the family is declining. My house is under construction*,* and one day*,* without my knowledge*,* all the flowers and plants on the roof were thrown away for construction work. I was not home at the time*,* and they did not even feel like asking me once. When I determined*,* it affected me*,* but when I asked about it*,* they thought I was overreacting”.* Reported by a 68-year-old male.

#### Common mental health problems in older adults

Older adults frequently experience a variety of psychological and emotional challenges, which significantly impact their life. They reported difficulties such as forgetfulness, depression, and persistent low mood, which were often exacerbated by the loss of loved ones and the accompanying sadness.

Common concerns included boredom, a sense of laziness, anxiety, nervousness, and sleep disturbances. Loneliness, feelings of being unwanted, and inadequate family support contributed to a sense of helplessness. Furthermore, mental health issues are compounded by physical ailments, further complicating their overall situation and well-being.

#### Health-seeking behaviors

Participants described multiple ways to care and cope, organized into sub-themes.

##### Professional help-seeking

Only a few older adults indicated a reliance on medical professionals such as doctors, psychiatrists, psychologists, counsellors, neurologists, and general practitioners for professional help.

##### Informal and social support systems

Support was sought from diverse networks, including friends, family members, parents, children, spouses, and loved ones, highlighting the importance of interpersonal relationships.

##### Spiritual and self-coping strategies

Spiritual and self-support were emerged, referencing seeking solace from God, practising self-reliance, and the importance of companionship or solitude. A pattern was observed in their responses: females tended to be more inclined to share, whereas males leaned more toward self-healing.

*“I share everything with my children; they are my best friends*,* and it makes me feel good. I also have some friends in my neighborhood who are around my age*,* so I can talk freely with them. I enjoy worshipping God and often sit quietly in front of my home temple. It makes me feel peaceful.”* Said a 60-year-old woman.

#### Suggestions to improve mental health

Older adults proposed multiple strategies to improve mental well-being, which were organized into the following sub-themes:

##### Management of physical and mental well-being

Most participants emphasized that maintaining physical health was central to improving mental well-being. They reported that consuming nutritious food, engaging in regular physical exercise, adhering to prescribed medications, practising yoga, and accessing appropriate medical care helped them feel physically stronger, which in turn reduced psychological distress. Meditation, devotional activities, prayer, and maintaining a positive outlook on life were frequently mentioned as important coping strategies (Fig. 2).

##### Social support and emotional expression

Older persons emphasized the need to share concerns with trusted individuals such as family members, friends, and close companions. Quality time with the family and engaging in social interactions are some of the key features to deal with emotional isolation associated with loneliness among older individuals.

##### Access to welfare and mental health services

The importance of accessing government welfare programs, mental health services, and psychological counseling to manage emotional distress were recognized by only a few participants.

##### Recreational and leisure activities

Participation in recreational pursuits was believed to improve mental health by serving as a source of entertainment that distracted individuals from their stress. Participants mentioned activities such as traveling with family or friends, listening to music, reading books, developing hobbies, spending time in nature, and watching television as beneficial for relaxation and mood enhancement. Females, in particular, expressed interest in activities such as cooking, dance-related activities.

*“Meditation and Yoga help me to calm me down. Because if my physical health is fine*,* I will be at peace. Talking about my concerns with family members*,* chatting with friends*,* and spending time with my pets also help me feel better.”* Reported a 61-year-old female.

#### Expectations from digital interventions

Expectations regarding digital interventions were categorized into multiple sub-themes:

##### Health and mental health content needs

During discussions on the features of digital intervention, both older adults and caregivers expected health management tips for managing physical health issues, organ care, exercise tutorials, precautions for specific health conditions, dietary guidelines, age-related awareness, mental healthcare advice, and sleep hygiene tips.

##### Access to health service and emergency support

Most caregivers emphasized the importance of incorporating medical facilities within the application, such as access to ambulances, helpline services, or connections to nearby medical facilities.

##### Social connectivity and emotional support

Suggestions made include music-based relaxation options and chat functionalities that would allow interaction with peers, which will in turn reduce the loneliness and promote social engagement.

##### Usability and accessibility of the digital intervention

Elements such as user-friendly design, facial recognition for log-in to accommodate those who may forget passwords, voice commands, large font sizes, regional language options, and intuitive prompts were considered crucial for ensuring the accessibility and effectiveness of the intervention (Supplementary Fig. 1).

### Perspective of the experts

#### Factors involved in healthy aging

Experts described healthy aging as a multidimensional process shaped by interconnected biological, psychological, and social factors. Their perspectives were organized into the following sub-themes:

##### Biological and functional health maintenance

All experts emphasized the importance of maintaining physical health through balanced nutrition, regular physical activity, preservation of functional ability, appropriate medication use, and routine health check-ups. Preventive care and early identification of health conditions were viewed as essential for sustaining independence in older age.

##### Psychological well-being, social engagement and family support

Most experts highlighted the role of emotional regulation, stress management, and cognitive engagement in preventing mental health decline and promoting overall quality of life. Social participation and strong family support systems were considered critical determinants of healthy aging. Experts noted that meaningful social interactions, intergenerational relationships, and community engagement help reduce loneliness and enhance emotional well-being.

##### Financial security and post-retirement planning

Financial stability was repeatedly mentioned as a key enabler of healthy aging. Experts stressed the importance of post-retirement planning, pension security, and financial literacy in reducing stress and enabling access to healthcare and social resources.

##### Role of policy, infrastructure and health systems

Accessible healthcare services, social welfare schemes, safe public spaces, and transportation systems were viewed as essential structural supports that enable older adults to remain active and engaged (Fig. 2).

*“Aging is a natural and inevitable process that cannot be significantly altered or delayed through human intervention. It is best to accept aging as a law of nature and adapt to its changes in a holistic manner to maintain a productive life. Each stage of life comes with specific goals and obligations*,* which evolve with age*,* and it is important to acknowledge and adapt to these changes accordingly.”* Opined by a psychiatrist,

In the analysis of free listings, a balanced diet emerged as the most important factor (salience 12.01), followed by physical exercise (salience 8.01), the management of physical ailments (salience 7.12), social involvement (salience 6.72), and familial support (salience 6.15) (Supplementary Table 3). The components most frequently cited were a balanced diet (frequency 16), social involvement (frequency 14), familial support (frequency 11), physical exercise (frequency 10), and managing physical ailments (frequency 10) (Supplementary Fig. 2). Network analysis indicated that the factors most commonly linked to healthy aging were a balanced diet and social involvement, which appeared together nine times. This was followed by social involvement and physical exercise, as well as a balanced diet and physical exercise, both of which co-occurred eight times (Supplementary Fig. 3).

#### Factors contributing to mental health problems

Experts identified multiple, interrelated determinants contributing to mental health problems among older adults. These were organized into the following sub-themes:

##### Biological and lifestyle-related factors

Experts, including psychiatrists, emphasized age-related biological changes such as neural degeneration, reduced neuroplasticity, and other brain-related changes as important contributors to mental health problems. Poor dietary choices, excessive medication use, and physical inactivity or idleness, particularly following retirement were also identified as factors negatively affecting cognitive and emotional well-being.

##### Psychosocial and family related stressors

Psychosocial challenges were reported as major contributors to mental health difficulties. These included verbal abuse from family members, lack of empathy, family conflicts, separation from children, and inadequate emotional support. Experts highlighted that such experiences often result in loneliness, feelings of neglect, and emotional distress among older adults.

##### Social isolation, economic stress and limited awareness

Insufficient social interaction and limited social support networks were perceived to exacerbate mental health problems, especially among older adults experiencing loneliness and reduced community engagement. Additionally, experts noted that poor recognition of mental health symptoms and limited awareness of mental health disorders often delay help-seeking. Post-retirement stress and financial insecurity were also viewed as significant contextual factors that intensify psychological distress (Fig. 2).

*“Financial insecurity and physical limitations are major concerns. People often feel distressed when they compare their current abilities with what they could do when they were younger. This sense of loss and insecurity can lead to depression and anxiety. Social insecurities also play a role*,* as individuals may feel overlooked or undervalued by others.”* Opined a geriatric specialist.

#### Challenges faced by experts in treating mental health problems in older adults

Experts described multiple, interrelated challenges that hinder effective mental healthcare delivery for older adults.

##### Stigma, limited awareness and help-seeking barriers

Older adults often lacked knowledge about self-care, overlooked early warning signs of mental health problems, and demonstrated reduced help-seeking behavior. Many were unaware of available treatment facilities, and mobility limitations further restricted access to care.

##### Clinical and system-level constraints

Clinical complexity, particularly the presence of comorbidities and polypharmacy, posed significant challenges for treatment planning and management. Experts also highlighted systemic barriers, including language differences, financial constraints, inadequate psychosocial management, limited family or community support systems, and the scarcity of geriatric-specific clinics. These challenges were compounded by insufficient government policies for older adults, along with shortages of trained manpower, healthcare resources, and assistive devices.

##### Treatment acceptability and engagement challenges

Treatment delivery was further hindered by therapeutic resistance and low acceptability among some older adults. Experts reported issues such as rigidity in beliefs, geriatric maladaptation, limited intrinsic motivation, resistance to modern therapeutic approaches, and poor follow-up adherence.

*“As people age*,* their brains become more sensitive to medication side effects*,* especially with psychotropic drugs. This makes treating mental health issues in older adults more challenging. Additionally*,* many elderly people lack social support*,* with fewer family members available to take them to medical appointments or help them when they are sick.”* Opined by a psychiatrist,

#### Preventive measures taken by experts to combat mental health problems

Experts described a range of preventive strategies aimed at reducing the risk of mental health problems among older adults.

##### Preparedness, awareness, and early identification

All experts underscored the importance of financial security, retirement planning, and supportive infrastructure as foundational elements of mental well-being in later life. They emphasized individual responsibility in preparing for aging, increasing awareness of psychiatric conditions, anticipating social isolation, and recognizing early symptoms of mental health problems.

Timely help-seeking and proactive engagement with mental health services were considered essential preventive measures.

##### Lifestyle and promotion of health

All healthcare providers advocated lifestyle modifications, including regular physical activity, balanced nutrition, adequate sleep, and adherence to prescribed medical advice.

##### Social, cognitive, and psychological engagement

Maintaining social connectedness through family interactions, community participation, and involvement in age-appropriate activities was strongly encouraged. Experts also highlighted the benefits of cognitive engagement, spiritual practices, stress management techniques, behavioral modifications, and psychotherapy in enhancing resilience and promoting sustained mental well-being (Fig. 2).

*“To prepare for later stages of life*,* it is important to understand what challenges may arise and how to handle them. Taking care of one’s physical health*,* staying socially engaged*,* and maintaining family relationships can help. Eat well and stay healthy.”* Opined by a general physician.

### Triangulation of the perspectives of the beneficiaries and the experts

Overall, the findings demonstrate convergence between beneficiaries and experts in conceptualizing healthy aging and mental health as interlinked, multidimensional processes shaped by physical health, psychological well-being, social relationships, and contextual supports. Beneficiaries primarily articulated lived experiences, emphasizing functional independence, emotional balance, family support, coping practices, and day-to-day challenges related to health, finances, and changing family roles. Experts complemented these narratives by situating individual experiences within broader biological processes, psychosocial stressors, health system limitations, and policy and infrastructural gaps. While beneficiaries highlighted immediate coping strategies and personal resilience, experts underscored preventive care, early identification, and system-level interventions as critical to sustaining well-being in later life. Taken together, the results indicate that effective promotion of healthy aging and mental well-being requires coordinated individual, familial, community, and health-system responses rather than isolated interventions.

## Discussion

Development of digital mental health services via technology requires an evidence-based approach that incorporates systems thinking and co-production and emphasizes stakeholder-centered design [41]. Different countries have adopted different activities to maintain healthy aging and reduce common mental health disorders [42–45]. The present study highlights the key points in creating a digital intervention for healthy aging and common mental health problems of older adults in the Indian sociocultural context. To the best of our knowledge, this digital intervention is the first of its kind for older adults in India. Creating a digital intervention requires collaboration with multiple parties and stakeholders [46]; this study highlights key points that have emerged from both beneficiaries and experts. Moreover, the study integrates perspectives from three stakeholder groups (older adults, caregivers, and experts), uses multiple qualitative methods (FGDs, IDIs, KIIs, free-listing), and directly informs the development of a culturally tailored digital intervention for older adults in India.

### Beneficiaries’ perspectives on mental health & healthy aging

Regular sleep, a balanced diet, exercise, stress management, meditation, and positive thinking emerged as key factors in maintaining healthy aging among beneficiaries. Additionally, strong family support, social connections, community involvement, and financial stability also emerged as important aspects. The experts also emphasized the importance of nutrition, exercise, functional ability, medication, and routine check-ups for sustaining healthy aging. They further underscored the significance of social engagement, family time, caregiver support, and mental well-being. A balanced diet, physical exercise, and the management of physical ailments were recognized as crucial through free listing. In another study, active social participation was found to be vital for the mental well-being of elderly people, with social activities potentially assisting them in coping with challenging conditions [4]. Factors such as involvement in household tasks, spiritual practices, and living arrangements also influence healthy aging outcomes [4]. In a study among older adults in India, engagement in physical activity and yoga, being younger, having good childhood health, and maintaining a healthy weight were found to be important determinants [47]. Another study from India reported that physical activity and employment status positively reinforce healthy aging [48]. Published studies have identified other contributing factors, such as being male, being married, being formally educated, having a high socioeconomic status, and living in urban areas [4, 47, 48]. The beneficiaries of the present study have also documented financial instability and societal challenges as barriers to achieving healthy aging, similar to other studies [47, 48]. Forgetfulness, depression, persistent low mood, and anxiety, along with nervousness and sleep disturbances, are common mental health problems, which is also supported by other studies [12, 49–51]. Moreover, mental health issues are intensified by physical conditions, complicating their overall wellness, which is also evident in other studies [10, 50]. The beneficiaries relied on medical professionals for expert care and sought support from family, friends, and close relationships for personal well-being. According to experts, older adults face stigma and lack awareness, leading to poor self-care, reluctance to seek help, and ignorance of warning signs. Mobility barriers, societal stigma, and comorbidities further hinder access to treatment. Both the beneficiaries and the experts believed that a healthy diet, regular exercise, medication, yoga, and medical care would improve physical health and positive mental well-being. Meditation, devotional activities, positive outlooks, family time, counseling, and recreational activities were also seen as essential for mental health. Working status and good physical health were positively associated with mental health-seeking behavior, as supported by other studies [14].

### Experts’ perspectives on mental health & healthy aging

From the experts’ perspective, increased awareness, early symptom recognition, timely treatment, and education on healthy aging were emphasized as crucial measures. The experts recommended lifestyle changes, such as exercise, nutrition, sleep, and adherence to medical advice, for overall well-being. They stressed the importance of social connectedness, community engagement, and therapy or behavioral adjustments when necessary. The WHO has also underscored the value of social support and encouragement for healthy habits, including maintaining a balanced diet, staying physically active, avoiding tobacco, and limiting alcohol consumption, as important strategies to enhance mental health well-being [15]. Although some of the domains mentioned, such as social connectedness and physical activity, are similar to those in Western countries, the ways these domains could be achieved are slightly different in the Indian context than in Western countries [42].

### Perspectives on digital intervention

Digital mental health services, delivered through mobile phones or web-based platforms, have the potential to increase the accessibility and reach of mental healthcare [52]. The digital intervention itself has several unique advantages, such as being accessible 24 × 7, allowing beneficiaries to use it at their convenience. Moreover, being polydigital in nature, it provides additional advantages of providing multiple interventions through the same platform [41]. For example, maintenance of sleep hygiene will be promoted while mindfulness and mood-logging support will also be provided for anxiety, depression, etc. Therefore, it is crucial to design a user interface to align with users’ needs while also optimizing it for retention and engagement.

The proposed digital self-help intervention under the Indo-Swedish collaborative project, will be delivered over four weeks. Each week will include condition-specific modules tailored to the respective domain. Suggestions from the stakeholders related to managing physical health challenges will inform the inclusion of tailored health management modules, including condition-specific exercise tutorials with appropriate safety precautions, dietary guidance, and age-appropriate health awareness content. Identified mental health needs will be addressed through dedicated sections offering psychoeducation, stress management strategies, and sleep hygiene guidance. Stakeholders’ emphasis on access to care will guide the integration of essential health service features within the application, such as emergency ambulance contacts, health helplines. To address social and emotional needs, the digital framework will encourage and facilitate peer interaction and reduce social isolation. In line with stakeholders’ emphasis on usability, the intervention will adopt a user-friendly, age-sensitive design with simple navigation and clear interfaces. Drawing on evidence that digital interventions enhance engagement [53], the framework will include self-tracking tools, goal-setting features, progress evaluation, and personalized feedback or recommendations. Collectively, these design elements will ensure alignment with stakeholder-identified needs and will support sustainable improvements in healthy aging and mental well-being among older adults. In this way, digital interventions will complement rather than replace existing mental health services, serving as a ‘digital glue’ to enhance the user experience and ensure the sustainability of services for future generations [41]. India’s “scan and share initiative” exemplifies the successful application of digital technologies, demonstrating the effectiveness of simple digital solutions in transforming healthcare delivery across the nation [54].

### Limitations

This study was conducted in two metropolitan cities in India with relatively higher socioeconomic status and literacy level, which may limit the application of the findings to older adults living in lower resource settings. The use of purposive and quota sampling might limit the generalizability of the findings. Data regarding age was not collected at the Bangalore study site due to unforeseen and unavoidable logistical constraints. This may restrict the characterization of the study population and affect the interpretation of the findings, particularly with respect to potential age-related differences across study sites. The selection of older adults who use smartphones excluded non-smartphone users, thereby omitting perspectives from less tech-savvy individuals and making the study more relevant to tech-savvy older adults. Furthermore, those who are not comfortable with smartphones may require alternative forms of support, making it essential to explore different intervention strategies for diverse groups.

## Conclusions

The present study highlights that healthy aging and mental well-being among older adults in the Indian context are shaped by an interrelated set of physical, psychological, social, and structural factors, as experienced and articulated by older adults, caregivers, and experts. Beyond identifying individual behaviors such as diet, exercise, and sleep, the findings underscore the centrality of family systems, social connectedness, financial security, and culturally embedded coping practices such as spirituality and meditation. Importantly, the study reveals that mental health in later life is not solely an individual-level concern but is deeply embedded within family dynamics, community structures, and access to supportive environments. These insights emphasize that digital interventions must go beyond information delivery to reflect sociocultural realities, address relational and contextual determinants, and remain sensitive to the lived experiences of older adults in India. While the findings provide valuable guidance for designing contextually relevant digital health interventions, they are grounded in urban, smartphone-using populations and therefore should not be overgeneralized to all older adults in India, particularly those in rural or low-resource settings. Future research should examine how such interventions can be adapted and evaluated across more diverse populations to ensure broader equity, accessibility, and effectiveness.

## Supplementary Information

Supplementary Material 1: Supplementary Table 1. Consolidated Criteria for Reporting Qualitative Research (COREQ) Checklist. Supplementary Table 2. Focus Group Discussion, In-depth Interview, and Key Informant Interveiw Guide. Supplementary Table 3. Total salience, Smith score, and frequency of various phrases derived from the free listing data of 28 participants. Supplementary Fig. 1 Cognitive map showing the codes, categories and themes of opinions regarding the features of the digital intervention platform of beneficiaries. Supplementary Fig. 2 Co-occurrence matrix of phrases in the free list. Supplementary Fig. 3 Network analysis representing the co-occurrence of various phrases in the free list.

## Data Availability

The datasets used and/or analysed during the current study consist of anonymized qualitative transcripts to protect participant confidentiality and are available from the corresponding author on reasonable request.

## Abbreviations

COREQ: Consolidated Criteria for Reporting Qualitative Research
FGD: Focus Group Discussion
IDI: In-depth Interview
KII: Key Informant Interview
UNFPA: United Nations Population Fund
WHO: World Health Organization
LMIC: Lower Middle-income Country
