# Supplementary material for: Stakeholder perspectives informing the design and development of a digital self-help intervention for healthy aging and mental well-being among older adults in urban India: a qualitative study
Source: BMC Geriatr. 2026 Aug 18;26:1070. doi: 10.1186/s12877-026-08107-0 (PMC13491730; doi:10.1186/s12877-026-08107-0)
Supplement: Supplementary file 1 [file 12877_2026_8107_MOESM1_ESM.docx]

**Supplementary tables & figures**

**Supplementary Table 1 Consolidated Criteria for Reporting Qualitative Research (COREQ) Checklist**

| **Item No** | | **Guide Questions/Description** | **Reported on Page #** |  |  |
| --- | --- | --- | --- | --- | --- |
| **Domain 1: Research team and reflexivity** | | | |  |  |
| **Personal Characteristics** | | | |  |  |
| 1. Interviewer/ facilitator | | Which author/s conducted the interview or focus group? | Page 6 |  |  |
| 2. Credentials | | What were the researcher’s credentials? E.g., PhD, MD | Page 6 |  |  |
| 3. Occupation | | What was their occupation at the time of the study? | Page 6 |  |  |
| 4. Gender | | Was the researcher male or female? | Page 5-6 |  |  |
| 5. Experience and training | | What experience or training did the researcher have? | Page 6 |  |  |
| **Relationship with participants** | | | |  |  |
| 6. Relationship established | | Was a relationship established prior to study commencement? | Page 5 |  |  |
| 7. Participant knowledge of the interviewer | | What did the participants know about the researcher? e.g. personal goals, reasons for doing the research? | Page 6 |  |  |
| 8. Interviewer characteristics | | What characteristics were reported about the interviewer/facilitator? e.g. Bias, assumptions, reasons and interests in the research topic | Page 6 |  |  |
|  | **Domain 2: study design** | | |  |  |
|  | **Theoretical framework** | | |  |  |
|  | 9. Methodological orientation and Theory | What methodological orientation was stated to underpin the study? e.g. grounded theory, discourse analysis, ethnography, phenomenology, content analysis | Page 7-19 |  |  |
|  | **Participant selection** | | |  |  |
|  | 10. Sampling | How were participants selected? e.g., purposive, convenience, consecutive, snowball | Page 5 |  |  |
|  | 11. Method of approach | How were participants approached? e.g., face-to-face, telephone, mail, email | Page 4-7 |  |  |
|  | 12. Sample size | How many participants were in the study? | Page 5-6 |  |  |
|  | 13. Non-participation Setting | How many people refused to participate or dropped out? Reasons? | Page 6 |  |  |
|  | 14. Setting of data collection | Where was the data collected? e.g., home, clinic, workplace | Page 4-5 |  |  |
|  | 15. Presence of nonparticipants | Was anyone else present besides the participants and researchers? | Page 6-7 |  |  |
|  | 16. Description of sample | What are the important characteristics of the sample? e.g. demographic data, date | Page 8 |  |  |
|  | **Data collection** | | |  | No |
|  | 17. Interview guide | Were questions, prompts, and guides provided by the authors? Was it pilot tested? | Page 6-7 |  |  |
|  | 18. Repeat interviews | Were repeat interviews carried out? If yes, how many? | Page 7 |  |  |
|  | 19. Audio/visual recording | Did the research use audio or visual recording to collect the data? | Page 7 |  |  |
|  | 20. Field notes | Were field notes made during and/or after the interview or focus group? | Page 8 |  |  |
|  | 21. Duration | What was the duration of the interviews or focus group? | Page 7 |  |  |
|  | 22. Data saturation | Was data saturation discussed? | Page 7 |  |  |
|  | 23. Transcripts returned | Were transcripts returned to participants for comment and/or correction? | Page 7 |  |  |
|  | **Domain 3: analysis and findings** | | |  |  |
|  | **Data analysis** | | |  |  |
|  | 24. Number of data coders | How many data coders coded the data? | Page 8 |  |  |
|  | 25. Description of the coding tree | Did the authors provide a description of the coding tree? | N/A |  |  |
|  | 26. Derivation of themes | Were themes identified in advance or derived from the data? | Page 8 |  |  |
|  | 27. Software | What software, if applicable, was used to manage the data? | Page 8 |  |  |
|  | 28. Participant checking | Did participants provide feedback on the findings? | N/A |  |  |
|  | **Reporting** | | |  |  |
|  | 29. Quotations presented | Were participant quotations presented to illustrate the themes/findings? Was each quotation identified? e.g., participant number | Page 9-18 |  |  |
|  | 30. Data and findings consistent | Was there consistency between the data presented and the findings? | Pg 8-19, 28, 30-34 |  |  |
|  | 31. Clarity of major themes | Were major themes clearly presented in the findings? | Page 8-19 |  |  |
|  | 32. Clarity of minor themes | Is there a description of diverse cases or a discussion of minor themes? | Page 8-19 |  |  |

**Supplementary Table 2 Focus Group Discussion, In-depth Interview, and Key Informant Interveiw Guide**

**Focus Group Discussion and In-depth Interview Guide**

Q.1 What is your opinion about aging healthy?

Q.2 In your opinion, what are the most important factors that will help for healthy aging?

Q.3 What are the some of the practices you follow at family & community level to maintain / achieve healthy aging?

Q.4 What are some of the problems / barriers faced by you at family & community level to achieve healthy aging (spirituality, diet, physical activity, music)?

Q.5 Then please suggest some of your opinion in solving those barriers / problems.

Q.6 How do you define your role in your family & community?

Q.7 What is your opinion regarding mental health problems among the elderly?

Q.8 In your opinion what are the factors contributing to these mental health problems (Problem: family conflict/ off sp. separation/debt of spouse/financial dependence)?

Q.9 In your opinion where should you go if you /someone have some mental health issues?

Q.10 What are the barriers in accessing these services (individual/family/community)?

Q.11 In your opinion, what might be the possible ways to improve / uplift mental health?

Q.12 What are the different activities you do on your smart phone / computer in day to day life?

Q.13 How do you use your smart phone / computer to sustain good health?

Q.14 In your opinion, how useful would be some intervention through your smart phone / computer in addressing healthy aging & or mental health?

Q.15 What features do you think would make a these type of interventions through phone / computer more accessible and user-friendly for older individuals?

**Key Informant Interview Guide**

Q.1 In your opinion, what are the most important factors that will help for healthy aging?

Q.2 What do you think are the most common mental health problems among the elderly population?

Q.3 What according to you can be the reasons for the mental health problems among this population?

Q.4 What kind of challenges do you face in handling/treating the health-related problems of older adults?

Q.5 What are the common solutions or preventive measures that can be taken by the elderly subjects to combat these mental health problems?

**Supplementary Table 3 Total salience, Smith score, and frequency of various phrases derived from the free listing data of 28 participants**

| PHRASES | TOTAL SALIENCE | SMITH'S SCORE | FREQUENCY |
| --- | --- | --- | --- |
| Balanced diet | 12.014 | 0.4290714286 | **16** |
| Physical exercise | 8.015 | 0.28625 | **10** |
| Taking care of Physical ailment | 7.129 | 0.2546071429 | **10** |
| Social involvement | 6.726 | 0.2402142857 | **14** |
| Family support | 6.159 | 0.2199642857 | **11** |
| Financial security | 6.151 | 0.2196785714 | **9** |
| Healthy lifestyle | 4.077 | 0.1456071429 | **5** |
| Social support | 2.928 | 0.1045714286 | **5** |
| Cognitive exercises | 2.851 | 0.1018214286 | **6** |
| Staying active | 2.749 | 0.09817857143 | **4** |
| Personal interests/hobbies | 2.684 | 0.09585714286 | **5** |
| Regular health check-up | 2.533 | 0.09046428571 | **6** |
| Interpersonal relationships | 2.43 | 0.08678571429 | **4** |
| Sleeping | 2.411 | 0.08610714286 | **5** |
| Positive attitude to life | 2.25 | 0.08035714286 | **4** |
| Self-awareness | 2.25 | 0.08035714286 | **3** |
| Congenial family environment | 2.222 | 0.07935714286 | **3** |
| Graceful self-acceptance of aging | 1.975 | 0.07053571429 | **4** |
| Elderly friendly environment | 1.91 | 0.06821428571 | **3** |
| Screening | 1.861 | 0.06646428571 | **4** |
| Physical activity | 1.75 | 0.0625 | **2** |
| Participation in daily household activities | 1.214 | 0.04335714286 | **2** |
| Health programmes targeting the elderly | 1.194 | 0.04264285714 | **2** |
| Spirituality | 1.144 | 0.04085714286 | **2** |
| Happiness | 1.083 | 0.03867857143 | **2** |
| Stress management | 1.008 | 0.036 | **3** |
| Liaisoning | 1 | 0.03571428571 | **1** |
| Structured daily activities | 0.966 | 0.0345 | **2** |
| Faith | 0.928 | 0.03314285714 | **1** |
| Staying with joint family | 0.916 | 0.03271428571 | **1** |
| Seeking help for mental well being | 0.904 | 0.03228571429 | **2** |
| Self-care | 0.888 | 0.03171428571 | **1** |
| Facilitating generational transition | 0.833 | 0.02975 | **2** |
| Good companionship | 0.75 | 0.02678571429 | **1** |
| Presence of social/public infrastructure (e.g. Lift, smooth roads) | 0.714 | 0.0255 | **1** |
| Quality of life | 0.6 | 0.02142857143 | **1** |
| Psychological stability | 0.6 | 0.02142857143 | **1** |
| Planning your retirement wisely/elderly phase | 0.566 | 0.02021428571 | **2** |
| Adequate management of comorbidities | 0.555 | 0.01982142857 | **1** |
| Good working environment | 0.555 | 0.01982142857 | **1** |
| Peaceful environment free from pollution | 0.541 | 0.01932142857 | **2** |
| Social acceptance | 0.5 | 0.01785714286 | **1** |
| Good settlement of children | 0.5 | 0.01785714286 | **1** |
| No addictions | 0.45 | 0.01607142857 | **2** |
| Grateful for life's experiences | 0.375 | 0.01339285714 | **1** |
| Disease free health | 0.375 | 0.01339285714 | **1** |
| Caregiver education | 0.333 | 0.01189285714 | **1** |
| Psycho-education to reduce stigma | 0.291 | 0.01039285714 | **2** |
| Psychotherapy to promote mental health | 0.271 | 0.009678571429 | **2** |
| Openness to accept emptiness | 0.25 | 0.008928571429 | **1** |
| Avoiding too much technology | 0.214 | 0.007642857143 | **1** |
| Activities with peer-group | 0.2 | 0.007142857143 | **1** |
| Acceptance of grief | 0.166 | 0.005928571429 | **1** |
| Pharmacological preventive methods | 0.142 | 0.005071428571 | **1** |
| Educating oneself on healthy aging | 0.125 | 0.004464285714 | **1** |
| Better access to healthcare and daily needs | 0.111 | 0.003964285714 | **1** |

**Supplementary Fig. 1 Cognitive map showing the codes, categories and themes of opinions regarding the features of the digital intervention platform of beneficiaries**

**Supplementary Fig. 2 Co-occurrence matrix of phrases in the free list**

**Supplementary Fig. 3 Network analysis representing the co-occurrence of various phrases in the free list**
